# Effect of alkaline microwaving pretreatment on anaerobic digestion and biogas production of swine manure

**DOI:** 10.1038/s41598-017-01706-3

**Published:** 2017-05-10

**Authors:** Tao Yu, Yihuan Deng, Hongyu Liu, Chunping Yang, Bingwen Wu, Guangming Zeng, Li Lu, Fumitake Nishimura

**Affiliations:** 1 Zhejiang Provincial Key Laboratory of Solid Waste Treatment and Recycling, College of Environmental Science and Engineering, Zhejiang Gongshang University, Hangzhou, Zhejiang 310018 P. R. China; 2 School of Civil and Building Engineering, Loughborough University, Loughborough, Leicestershire LE11 3TU United Kingdom; 3 College of Environmental Science and Engineering, Hunan University, Changsha, Hunan 410082 P. R. China; 4 Department of Environmental Engineering, Graduate School of Engineering, Kyoto University, C1-2-221, Nishikyo-ku, Kyoto 615-8540 Japan

## Abstract

Microwave assisted with alkaline (MW-A) condition was applied in the pretreatment of swine manure, and the effect of the pretreatment on anaerobic treatment and biogas production was evaluated in this study. The two main microwaving (MW) parameters, microwaving power and reaction time, were optimized for the pretreatment. Response surface methodology (RSM) was used to investigate the effect of alkaline microwaving process for manure pretreatment at various values of pH and energy input. Results showed that the manure disintegration degree was maximized of 63.91% at energy input of 54 J/g and pH of 12.0, and variance analysis indicated that pH value played a more important role in the pretreatment than in energy input. Anaerobic digestion results demonstrated that MW-A pretreatment not only significantly increased cumulative biogas production, but also shortened the duration for a stable biogas production rate. Therefore, the alkaline microwaving pretreatment could become an alternative process for effective treatment of swine manure.

## Introduction

Animal manure is one of the major wastes in many agricultural countries due to their intensive animal breeding industry, and it has become a big challenge that should be appropriately treated^1^. The increased and concentrated animal waste generated odour problem and contained pathogen which will threaten people’s health if not handled properly. Moreover, it contained nutrients and heavy metals which will impact the quality of surface and ground water if they are discharged directly^2^.

Traditionally, animal manure is treated by anaerobic digestion. The results showed pathogens were destroyed and wastes were stabilized through the process. also, it generated biogas that can be used for daily activities^3, 4^. However, the high content of fiber in animal manure was limited the efficiency of anaerobic digestion which cannot be well utilized by anaerobic bacteria^5^. Thus, enhanced biogas production by anaerobic digestion has been paid great attention, and substrate optimization has been focused.

Carlsson *et al*.^6^ mentioned that pretreatment of manure to break down its structures could be effective for the enhancement of anaerobic digestion. Generally, pretreatment can be roughly divided into three groups, physical pretreatment, chemical pretreatment, and biological pretreatment^7^. Among them, thermo-chemical pretreatment is a main method used in current studies^8, 9^. Alkaline is a simpler and easier handling chemical pretreatment method compared with others, especially when combined with thermal effect. Currently, MW pretreatment is a favorable thermal pretreatment method. Compared with traditional heating techniques, MW has the advantages of shorter reaction time and lower energy consumption^10^. Furthermore, microwave pretreatment when combined with other technologies shows better degradation performance^11^.

This study was used microwave as pretreatment under alkaline condition (MW-A). By combination of the two different technologies, the performance of pretreatment was significantly improved^12^, Currently, MW was successful used in activated sludge and anaerobic digestion pretreatment^13, 14^. It has great potential to apply in animal manure. However, few research has been reported on MW pretreatment of animal manure. This study was to investigate the mechanism of MW-A by treating animal manure and consider combination effects of microwaving duration, microwave power, and alkaline dosage. Furthermore, this study deeply researched the effects of combining pretreatment of alkaline and microwave on anaerobic digestion at various values of pH and ammonium nitrogen (AN) concentrations. RSM was involved which is a systematic research strategy for studying the interaction of various parameters effect using statistical methods^14^. The main objective of this study was to evaluate the effect of pH and energy input on swine manure pretreatment and the effects of pH and AN concentration on the consequent anaerobic digestion of swine manure with various pretreatment by RSM.

## Results

### Optimization for manure pretreatment with RSM

Together with the response values, the complete design matrixes are shown in Table 1. The highest DD reached 63.91% in this study. The corresponding second order polynomial fitting equation was as follows:

$${\rm{DD}}( \% )={\rm{37.88}}+15.43{x}_{1}+8.48{x}_{2}-5.57{x}_{1}^{2}+12.08{x}_{2}^{2}-5.76{x}_{1}{x}_{2}$$

Whether the model was proper or not was checked by the analysis of variance(ANOVA). The model was calculated by F-value and P-value (Prob > F). When P-value is less than 0.05 which is means that the model is highly significant. The smaller value shows the greater effect^15^. The ANOVA for the quadratic of model for disintegration degree of manure is listed in Table 2. In the study, the P-value of model was less than 0.05 and the Lack of Fit Test was not significant, which indicated that the model had a favorable fitting degree.

**Table 1:** Experiment design matrix for combined Alkaline microwaving pretreatment.

Run	Coded variables		Experimental variables		DD (%)
	x_1_	x_2_	x_1_	x_2_(J/g)	
1	0	0	10	54	36.40
2	−2	0	8	54	33.05
3	1	1	11	72	54.98
4	−1	1	9	72	42.34
5	0	−2	10	18	24.13
6	0	0	10	54	33.42
7	2	0	12	54	63.91
8	0	2	10	90	37.14
9	1	−1	11	36	45.32
10	−1	−1	9	36	27.11

**Table 2:** ANOVA for a quadratic response surface model.

Source	Squares	df	Square	Value	Prob > F	
Model	1270.27	5	254.05	11.31	0.0178	significant
A-x_1_	714.1	1	714.1	31.79	0.0049	
B-x_2_	215.99	1	215.99	9.61	0.0362	
x_1_x_2_	7.76	1	7.76	0.35	0.5884	
x_1_^2^	151.41	1	151.41	6.74	0.0603	
x_2_^2^	34.43	1	34.43	1.53	0.2834	
Lack of Fit	85.42	3	28.47	6.41	0.2807	not significant
R^2^ = 0.9339						

**Figure 1:**
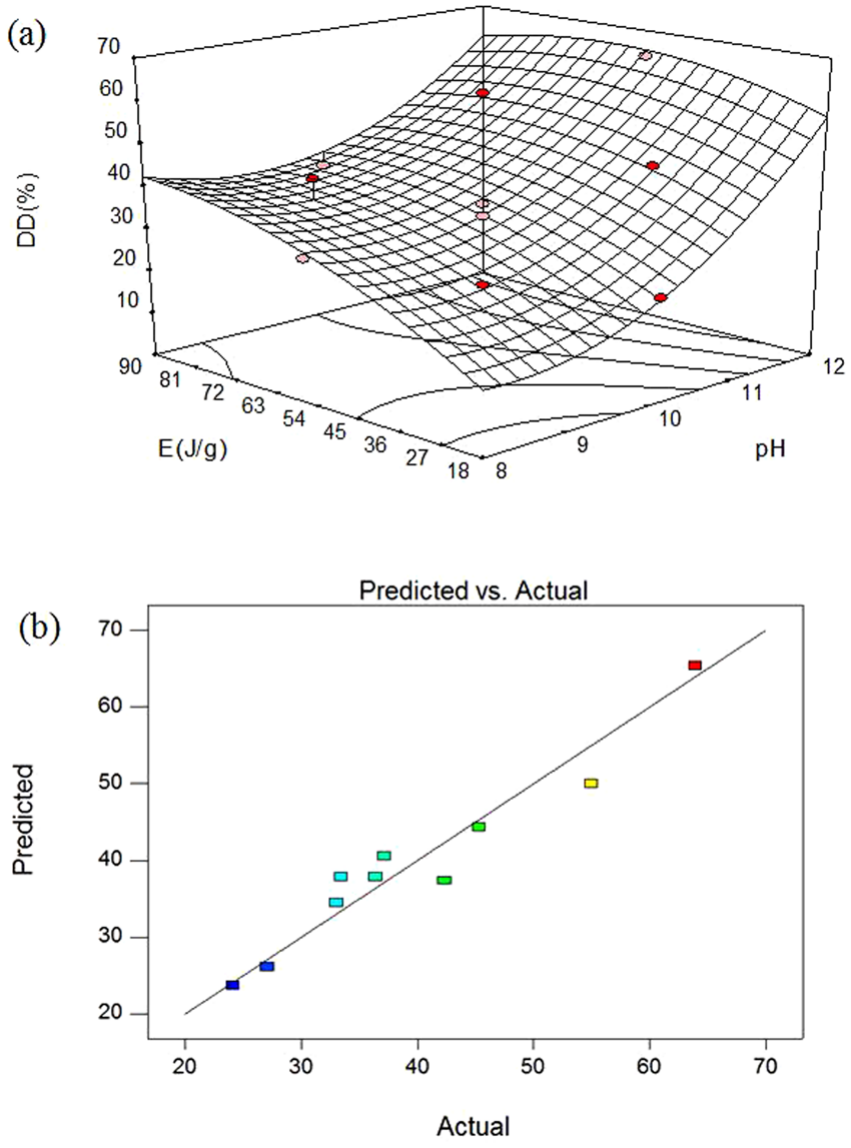
Analysis of Response surface methodology: interactive effects of pH and E on DD. (**a**) 3D response surface; (**b**) Verification result of RSM predicts and determined.

Figure 1(a) showed the effect of pH and energy on DD. The trend of DD of manure was increase to peak and dropped after. The peak of DD was 63.91% when energy input was 54 J/g and pH was 12.

In order to investigate model accuracy and practicability, the predicted value and actual value were compared in Fig. 1(b). The maximum experimental DD of manure was 63.91% while the predicted value was 62.75%, and the absolute error was less than 2%. The correctness and validation of the simulation model is demonstrated.

As a conclusion, the optimum conditions for DD was 54 J/g for energy input with stronger alkaline condition (pH 12).

### Changes of pH on biodegradation

**Figure 2:**
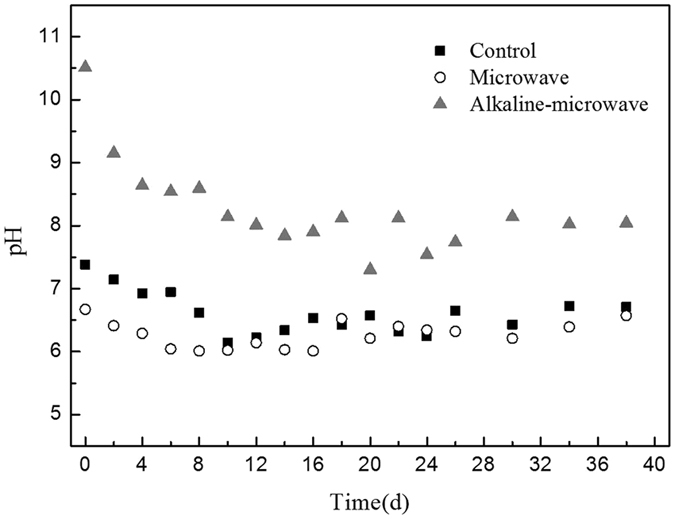
Changes of pH during the anaerobic digestion after different pretreatments.

The two groups of manure samples were pretreated by MW with 300 W power and reaction time of 180 s, which was based on previous conclusion. Figure 2 presented the changes of pH during the anaerobic digestion.

The results showed that during anaerobic digestion, The MW-A remained weak alkaline environment, while the microwaving pretreatment and control group turn to acidic.

### Changes of protein concentration on biodegradation

**Figure 3:**
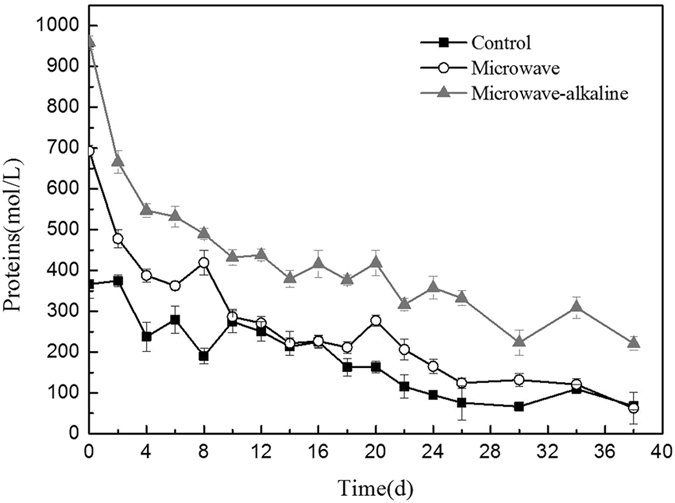
Changes of soluble organics during the anaerobic digestion after different pretreatments.

As shown in Fig. 3, after MW pretreatment, the protein concentration was rapidly reduced at the first four day, and then the rate of the degradation decreased 8 days later. The rates were gradually decreased for MW and MW-A groups. The trend of control group was slightly reduced in the whole process, compared others. The protein concentration of MW-A pretreatment was higher than other two groups.

The protein concentration after alkaline microwaving pretreatment remained a relative high level during the whole anaerobic digestion.

### Changes of ammonium nitrogen (AN) concentration on biodegradation

**Figure 4:**
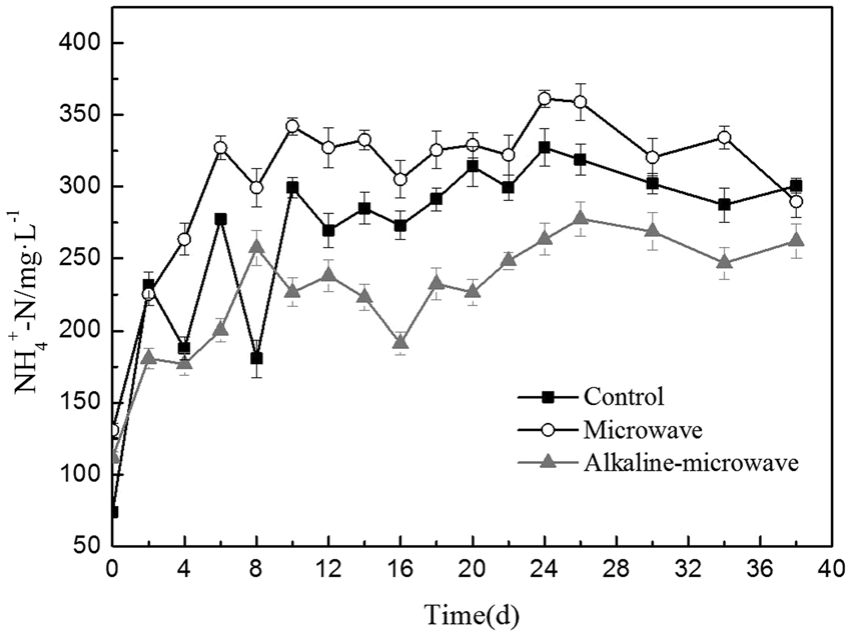
Changes of ammonium nitrogen during the anaerobic digestion after different pretreatments.

The AN concentration during the anaerobic digestion by different pretreatments was recorded and shown in Fig. 4. As it can be seen in Fig. 4, the content of AN were increased for three groups The MW group was the highest amount of AN concentration, and then in control group, the MW-A group was the lowest one. Figure S2 provided a basis for above changes of AN, and it presented that the dissolution rate of ammonia decreased with the increasing of pH. When pH was more than 8, AN density made little change with the increasing of the reaction time. By the stronger alkaline condition, the AN hardly released from manure.

### Biogas accumulation

**Figure 5:**
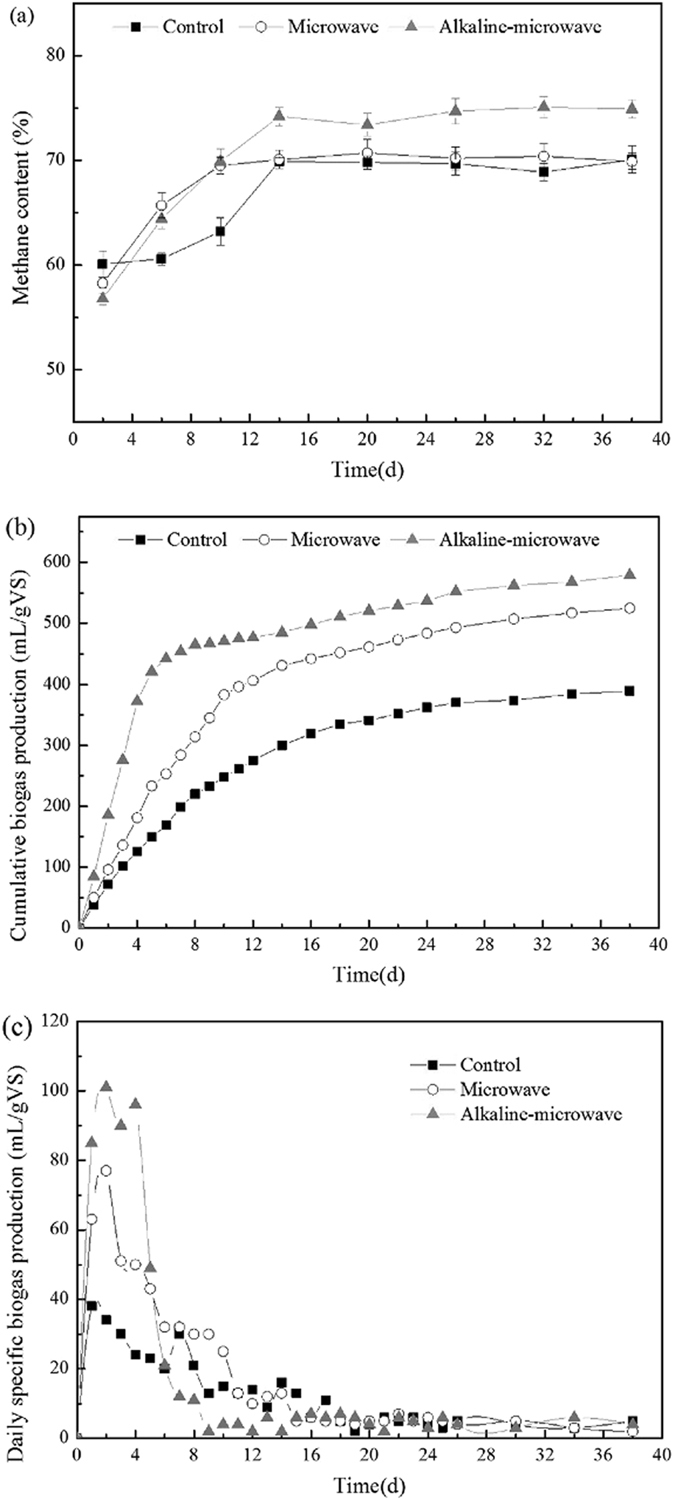
Methane content and cumulative biogas production of manure after different pretreatments. (**a**) Methane content; (**b**) Cumulative biogas production; (**c**) Daily specific production.

Figure 5(a) showed the methane content by anaerobic digestion through different pretreatment. The MW-A group reached 75%, and others lower around 5%.

The effect of different pretreatments on total biogas production is shown in Fig. 5(b). The cumulative biogas production was the highest in MW-A pretreatment group, and then in MW only group, and it was the lowest in control group. The biogas production rate for MW-A, MW and control groups began to stabilize in 8 days, 14 days and 14 days, respectively. The daily biogas production after different pretreatments is shown in Fig. 5(c), it showed that there was little biogas production after 16 days, 18 days, 18 days, respectively. So the duration for a stable biogas production rate on three groups was 8 days, 4 days, 4 days, respectively.

The results showed MW-A pretreatment was not only effectively increased the total biogas production, but also shortens the time for a stable biogas production rate.

## Discussion

Table 1 shows that through the MW-A pretreatment, the DD of manure was increased significantly. This could due to microwave radiation break down the complex structure of the polymer substances, and it can make proteins and sugars enter into a soluble phase^16^, meanwhile alkaline effectively solubilize particulate organic matter and improve the digestibility^17^. There was a positive correlation among the DD of manure, the alkaline degree and the energy input.

According to Table 2, x_1_ and x_2_ were significant influence factors, which means that pH and energy could significantly improve the DD of manure. Within the two parameters, pH was more important than energy input which reported by Doğan and Sanin^18^. The 3D response surfaces plot also revealed that the DD of manure increased along with enhancing energy input and alkaline degree (see Fig. 1(a)). The results confirmed that the high pH value was benefit for pretreatment. For example, the energy input remained the same but pH levels increased from 8 to 12, the DD rose from 33.05% to 63.91%. On the contrary, the DD had no significant difference when pH remaining at 10 and energy input enhanced from 54 J/g to 90 J/g, which got 36.42% and 37.14%, respectively. The comparison of predicted value and actual value (Fig. 1(b)) also indicated that the alkaline microwaving pretreatment could promote the disintegration degree of manure.

In general, the appropriate pH is 6.8–7.2 for completely mixed digesters^19^. Therefore, if the initial pH was higher than 10 (NaOH dose was 0.1 mol/L or 0.2 g/g TS), the anaerobic digestion process may have been obliterated^20^. In anaerobic digestion process, organic acids were accumulated when the rate of hydrolysis and acidification stages were over methane-producing stage, and this would cause pH decline. On the contrary, pH would enhance when organic acids could not effectively accumulate with similar rate of two stages^21^. Methanogens showed more sensitive to pH than acid formation bacteria^22^, though few acidophilic methanogens had been found^23^, most of which could only survive in neutral or weak alkaline environment, the accumulation of organic acids in the process of anaerobic digestion would inhibit the growth of methanogens^24^. In Fig. 2, only the combined pretreatment group remained weak alkaline environment for a long time, which implied that methanogens was always the dominant microflora through alkaline-microwave pretreatment group.

The protein is the main constituent of manure, and the manure proteins converted to soluble proteins and in hydrolysis and acidification process, respectively. The soluble organics were mainly produced by microwave effect, and absorbed by acid formation bacteria rapidly^25^. After burning up initial organics, the hydrolysis of manure began to release soluble organic gradually. The protein content of combined alkaline microwaving pretreatment was relatively higher in Fig. 3, which implied that excess acidity alkali promoted manure’s hydrolysis^26^.

The AN mainly comes from protein and other nitrogenous organics’ degradation:

$$\begin{array}{c}{{\rm{RCHNH}}}_{2}{\rm{COOH}}+2{{\rm{H}}}_{2}{\rm{O}}\to {\rm{RCOOH}}+{{\rm{CO}}}_{2}+{{\rm{H}}}_{2}+{{\rm{NH}}}_{3}\\ {{\rm{NH}}}_{3}+{{\rm{H}}}_{2}{\rm{O}}\to {{\rm{CO}}}_{2}+{{\rm{NH}}}_{4}+{{\rm{HCO}}}_{3}\end{array}$$

According the results presented in Fig. 4, the pretreatment group’s AN density of microwave was greater than the control group, the reason may lie in that there was more protein hydrolysis of AN after pretreatment for microwave. Many earlier studies had investigate that pH and AN concentration could affected the methane production^27^. By adjusting pH to 8.0, a stable digestion of a synthetic acetic acid substrate at inhibitory TAN (total AN concentration) levels of 500 mg/l was maintained^27^. The high-solids sludge digester could be operated satisfactorily at pH of 9.0 and AN concentration of 900 mg/l^28^. The methane yields in mesophilic Anaerobic Digester were optimum at pH of 7.9 and 3300 mg TAN/l concentration. However, as TAN went up to 5500 mg/l, 50% reduction in methane production was observed^29^. But few studies showed that the relationship between pH and AN concentration. Figure S2 presented that the dissolution rate of ammonia decreased with the increasing of pH, so although the protein concentration was the highest after combined pretreatment, because of the weak alkaline environment, which resulted in the low activity of enzymes relative, and the degradation of protein and other nitrogenous organics became more difficult. As a results, the AN content was lower in the digester.

The result of Fig. 5(a) shows that there is a linear correlation between solubilisation and biogas yield^30^, higher energy increases biogas yield even after reaching the boiling point in pretreatment^31^. The disintegration degree of manure was the highest after combining alkaline microwaving pretreatment, the substrate of methanogens was plenty and the hydrolysis rate grew rapidly in the beginning of the anaerobic digestion^5^.

This study showed MW-A group achieved the highest total biogas production than others. The biogas production mainly relies on methanogen. However, the methanogen was inhibited when excess concentration of AN existed^32^. Even though ammonia is an essential nutrient for bacterial growth, it may inhibit methanogenesis during anaerobic digestion process if it is available at high concentrations^27^. The MW-A pretreatment seemed to improve the recovery speed and stability of an ammonia-inhibited biogas digester fed with cattle manure^33^. The AN concentration was lower and pH presented weak alkalinity during anaerobic digestion process, which was suitable for methanogen growth and consequently improved biogas production. This study demonstrated that the MW-A pretreatment improved performance of anaerobic digestion by increasing biogas amount and accelerating the reaction rate.

## Methods

### Apparatus

The device of microwave reaction includes the microwave power system, reactor chamber and condenser. The microwave power system whose brand is MY1000S was provided by Huiyan Microwave Corporation (Nanjing, Jiangsu, China). The power of the microwave reaction ranges from 0 to 1000 W. The reaction time was controlled by a timer. The microwave reactor schematic is shown in Fig. S1.

### Manure

The swine manure for the experiment was obtained from the rural family swine farms. Fresh manure was smashed and filtered through a 0.45 mm sizing screen, and then refrigerated at 4 °C before study. The characteristics of the manure were listed in Table 3.

**Table 3:** Characteristics of swine manure used in this study.

pH	TCOD (mg/g TS)	SCOD (mg/g TS)	TS(%)	VS(%)
7.14 ± 0.26	208.4 ± 16.3	50.39 ± 0.82	6.02 ± 0.12	4.26 ± 0.06

### Microwaving pretreatment

The alkaline microwaving pretreatment for manure disintegration was conducted as follows: The pH value of 100 ml manure was firstly adjusted to 8.0, 9.0, 10.0, 11.0 and 12.0, respectively, by adding 1.0 M sodium hydroxide (NaOH) or 1.0 M hydrochloric (HCl), and the desired pH values were kept with ±0.1 unit fluctuations. Previous research has shown that the rate of hydrolysis and release rate of protein would arrive at maximum when microwave power reached 300 W, and the earlier study has also reached the same conclusion^26^. So the manure in a quartz reactor was immediately heated in microwave reaction chamber with a microwave power of 300 W. The energy input was set as 18, 36, 54, 72, or 90 J/g by altering the irradiation time. Energy (E) was determined by microwave power (P), microwave time (t), manure solution volume (V), and manure solution volume density (ρ):

1 $${\rm{E}}({\rm{J}}/{\rm{g}})=\frac{{\rm{P}}\times {\rm{t}}}{{\rm{V}}\times \rho }$$

Each sample was analyzed in triplicate, and the average values were determined for each set.

### Anaerobic digestion experiment

Anaerobic digestion experiments were conducted in three glass amber bottles. The effective volume of reaction system is 500 mL each. Every reactor contained a volume of 300 mL manure. The control group was set by adding untreated manure to one reactor and keeping the pH value at 7.0. The other two experimental groups were added with manure pretreated by microwave power of 300 W and reaction time of 180 s, which was based on the result of RSM. One experimental group’s pH value was adjusted to 12.0 by 1 M NaOH or 1 M hydrochloric (HCl), the other was kept at 7.0. The reactors were placed in Stirring hot plate (100 rpm, 35 ± 1 °C) for 40 days, and the gas production was recorded once a day. In order to maintain strict anaerobic condition, oxygen was removed by nitrogen gas sparging before fermentation.

### Analysis

Soluble chemical oxygen demand (SCOD), total chemical oxygen demand (TCOD), pH, total solid (TS), and volatile solid (VS) were measured according to the Standard Methods. Soluble protein was determined by the Folin phenol method with bovine serum albumin (BSA) as standard^34^. AN was determined by Nessler reagent spectrophotometry^35^. Biogas production was measured with a wet gas meter. Methane was detected by a gas chromatograph (GC7890A, Agilent Technologies, Inc., USA).

### Response surface methodology (RSM)

RSM is a sensitivity analysis method, which can improve the output of the model and its related input variables. Compared with orthogonal test, RSM can analyze every level of the experiment continuously in the optimization process, while orthogonal test can only study independent data points.

In this study, the experimental design was to combine a central composite design (CCD) with Design-Expert, a software used for data collection and analysis, which was used to research the effect of two independent variables: pH and energy (E). These two variables’ respective ranges were chosen in Table 1.

The target response was the disintegration degree (DD) of manure, which could be calculated by the following formula:

2 $${\rm{Disintegration}}\,\mathrm{degree}( \% )=\frac{{\mathrm{SCOD}-\mathrm{SCOD}}_{{\rm{0}}}}{{{\rm{TCOD}}}_{{\rm{0}}}{-\mathrm{SCOD}}_{{\rm{0}}}}\times {\rm{100}}$$

where SCOD is the SCOD of the pretreated manure, and SCOD0, TCOD0 is the SCOD, TCOD of the untreated manure, respectively.

The experimental data was fitted by a second-degree polynomial equation:

3 $${\rm{Y}}={{\rm{\beta }}}_{{\rm{0}}}+{{\rm{\beta }}}_{{\rm{1}}}{x}_{1}+{{\rm{\beta }}}_{{\rm{2}}}{x}_{2}+{{\rm{\beta }}}_{{\rm{11}}}{x}_{1}^{2}+{{\rm{\beta }}}_{{\rm{22}}}{x}_{2}^{2}+{{\rm{\beta }}}_{{\rm{12}}}{x}_{1}{x}_{2}$$

Y is the response variable, x_1_ and x_2_ are the coded variables. The model prediction is determined by a series of regression coefficient β, including central point β_0_, linear coefficients β_1_, β_2_, interaction coefficient β_11_ and quadratic coefficients β_11_, β_22_.

### Ethical statement

This article does not contain any studies with human participants or animals performed by any of the authors.

## Electronic supplementary material

Supplementary Information

## References

[1] Deng Y, Wheatley A (2016). Wastewater treatment in Chinese rural areas. Asian Journal of Water. Environment and Pollution.

[2] Xu JL, Adair CW, Deshusses MA (2016). Performance evaluation of a full-scale innovative swine waste-to-energy system. Bioresource Technology.

[3] Luo L (2016). Nutrient removal and lipid production by Coelastrella sp. in anaerobically and aerobically treated swine wastewater. Bioresource Technology.

[4] Wu, S. H. *et al*. Effects of C/N ratio and bulking agent on speciation of Zn and Cu and enzymatic activity during pig manure composting. *International Biodeterioration & Biodegradation* (2016).

[5] Jin Y, Hu Z, Wen Z (2009). Enhancing anaerobic digestibility and phosphorus recovery of dairy manure through microwave-based thermo-chemical pretreatment. Water Research.

[6] Carlsson M, Lagerkvist A, Morgan-Sagastume F (2012). The effects of substrate pre-treatment on anaerobic digestion systems: a review. Waste Management.

[7] Wen S (2016). Treatment of anaerobically digested swine wastewater by Rhodobacterblasticus and Rhodobactercapsulatus. Bioresource Technology.

[8] Guo JY, Yang CP, Peng LY (2014). Preparation and characteristics of bacterial polymer using pre-treated sludge from swine wastewater treatment plant. Bioresource Technology.

[9] Guo JY, Yang CP, Zeng GM (2013). Treatment of swine wastewater using chemically modified zeolite and bioflocculant from activated sludge. Bioresource Technology.

[10] Motevali A (2014). M. H. Comparison of energy parameters in various dryers. Energy Conversion and Management.

[11] Yeneneh AM, Kayaalp A, Sen TK, Ang HM (2015). Effect of microwave and combined microwave-ultrasonic pretreatment on anaerobic digestion of mixed real sludge. Journal of Environmental Chemical Engineering.

[12] Cheng XY, Liu CZ (2010). Enhanced biogas production from herbal‐extraction process residues by microwave-assisted alkaline pretreatment. Journal of Chemical Technology and Biotechnology.

[13] Ebenezer AV (2015). Effect of deflocculation on the efficiency of low-energy microwave pretreatment and anaerobic biodegradation of waste activated sludge. Applied Energy.

[14] Yang CP (2016). Catalytic oxidative desulfurization of gasoline using catalyst of molybdenum supported on 4A molecular sieve. Separation and Purification Technology.

[15] Li CL (2010). Kinetics model for combined (alkaline+ultrasonic) sludge disintegration. Bioresource Technology.

[16] Eskicioglu C, Kennedy KJ, Droste RL (2006). Characterization of soluble organic matter of waste activated sludge before and after thermal pretreatment. Water Research.

[17] Li XJ, Zhang RH, Pang YZ (2008). Characteristics of dairy manure composting with rice straw. Bioresource Technology.

[18] Doğan I, Sanin FD (2009). Alkaline solubilization and microwave irradiation as a combined sludge disintegration and minimization method. Water Research.

[19] Lay JJ (1997). Analysis of environmental factors affecting methane production from high-solids organic waste. Water Science and Technology.

[20] Li H, Li C, Liu W, Zou S (2012). Optimized alkaline pretreatment of sludge before anaerobic digestion. Bioresource Technology.

[21] Kang XR (2011). Effect of initial pH adjustment on hydrolysis and acidification of sludge by ultrasonic pretreatment. Industrial & Engineering Chemistry Research.

[22] Garcia JL, Patel BK, Ollivier B (2000). Taxonomic, phylogenetic, and ecological diversity of methanogenic Archaea. Anaerobe.

[23] Bräuer SL (2006). Characterization of acid-tolerant H2/CO2-utilizing methanogenic enrichment cultures from an acidic peat bog in New York State. FEMS Microbiology Ecology.

[24] Chen Y, Cheng JJ, Creamer KS (2008). Inhibition of anaerobic digestion process: a review. Bioresource Technology.

[25] Hu Z, Wen Z (2008). Enhancing enzymatic digestibility of switchgrass by microwave-assisted alkali pretreatment. Biochemical Engineering Journal.

[26] Yang Q (2013). Improving disintegration and acidification of waste activated sludge by combined alkaline and microwave pretreatment. Process Safety and Environmental Protection.

[27] Yenigün O, Demirel B (2013). Ammonia inhibition in anaerobic digestion: a review. Process Biochemistry.

[28] Lay JJ, Li YY, Noike T (1998). The influence of pH and ammonia concentration on the methane production in high-solids digestion processes. Water Environment Research.

[29] Westerholm M, Müller B, Arthurson V, Schnürer A (2011). Changes in the acetogenic population in a mesophilic anaerobic digester in response to increasing ammonia concentration. Microbes and Environments.

[30] Passos F, Solé M, García J, Ferrer I (2013). Biogas production from microalgae grown in wastewater: effect of microwave pretreatment. Applied Energy.

[31] Sólyom K, Mato RB, Pérez-Elvira SI, Cocero MJ (2011). The influence of the energy absorbed from microwave pretreatment on biogas production from secondary wastewater sludge. Bioresource Technology.

[32] Rajagopal R, Massé DI, Singh G (2013). A critical review on inhibition of anaerobic digestion process by excess ammonia. Bioresource Technology.

[33] Nielsen HB, Ahring BK (2007). Effect of tryptone and ammonia on the biogas process in continuously stirred tank reactors treating cattle manure. Environmental Technology.

[34] Lowry OH, Rosebrough NJ, Farr AL, Randall RJ (1951). Protein measurement with the Folin phenol reagent. J BiolChem.

[35] Krug F, Růžička J, Hansen E (1979). Determination of ammonia in low concentrations with Nessler’s reagent by flow injection analysis. Analyst.

