# Supplementary material for: Effect of alkaline microwaving pretreatment on anaerobic digestion and biogas production of swine manure
Source: Sci Rep. 2017 May 10;7:1668. doi: 10.1038/s41598-017-01706-3 (PMC5431765; doi:10.1038/s41598-017-01706-3)
Supplement: Supplementary file 1 — Supplementary Information [file 41598_2017_1706_MOESM1_ESM.pdf]

Scientific Reports

Effect of alkaline microwaving pretreatment on anaerobic digestion and biogas production of swine manure

Tao Yu[1,†], Yihuan Deng[2,†], Hongyu Liu[3,†], Chunping Yang[1,3,*], Bingwen Wu[1], Guangming Zeng[3], Li Lu[1], Fumitake Nishumura[4,*]

1. Zhejiang Provincial Key Laboratory of Solid Waste Treatment and Recycling, College of Environmental Science and Engineering, Zhejiang Gongshang University, Hangzhou, Zhejiang 310018, P. R. China
2. Civil and Building Engineering, Loughborough University, Loughborough, Leicestershire LE11 3TU, United Kingdom
3. College of Environmental Science and Engineering, Hunan University, Changsha, Hunan 410082, P. R. China
4. Department of Environmental Engineering, Graduate School of Engineering, Kyoto University, C1-2-221, Nishikyo-ku, Kyoto 615-8540, Japan

* Corresponding authors. Email: yangcp@zjgsu.edu.cn (C. Yang), nishimura.fumitake.3n@kyoto-u.ac.jp (F. Nishimura)

† These authors contribute to this paper equally.

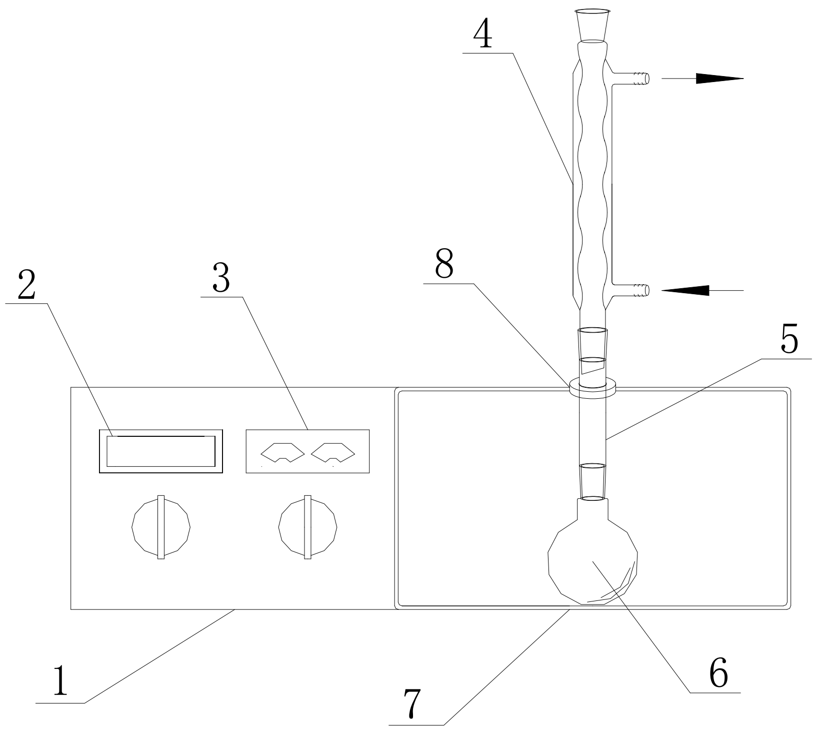

Fig. S1. The reactor schematic (1. microwave power supply; 2. timing device; 3. power regulator; 4. condenser pipe; 5. connecting pipe; 6. quartz flask; 7. reaction chamber; 8. cutoff waveguide)

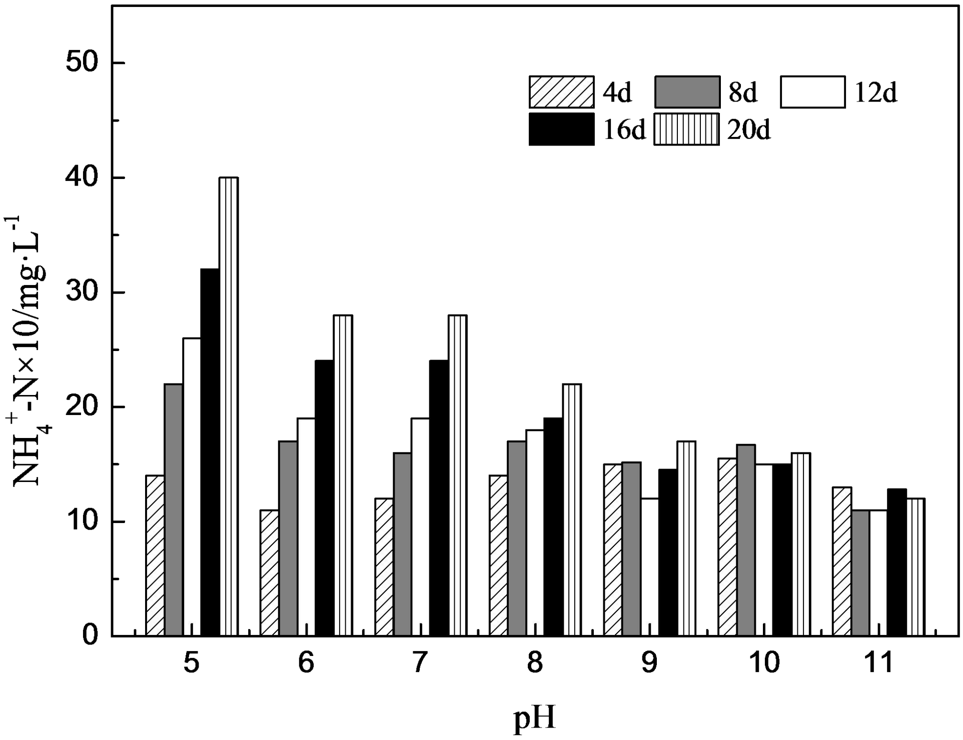

Fig. S2. Effect of pH on a ammonium nitrogen concentration during anaerobic digestion in 20 days. The results showed that the smaller of pH, more helpful for dissolving.
